# The Neuroanatomical Correlates of Visceral Pain: An Activation Likelihood Estimation Meta-Analysis

**DOI:** 10.3390/brainsci15060651

**Published:** 2025-06-17

**Authors:** Christoph Müller, Hagen Maxeiner

**Affiliations:** 1 Department of Internal Medicine, Lahn-Dill-Kliniken, 35578 Wetzlar, Germany; 2 Department of Internal Medicine, University of Marburg, 35037 Marburg, Germany; 3 Department of Anaesthesiology, Intensive Care Medicine and Pain Therapy, Lahn-Dill-Kliniken, 35578 Wetzlar, Germany

**Keywords:** visceral pain, activation likelihood estimation meta-analysis, functional neuroimaging, interoception

## Abstract

**Background:** Acute visceral pain is among the most common symptoms of patients seeking in-hospital treatment and is related to various thoracic, abdominal, and pelvic diseases. It is characterized by distinguishable sensory qualities and can be described on a sensory-discriminative and affective-motivational level. These sensory qualities correlate with the activation of cerebral areas involved in the neuronal processing of visceral pain and can be visualized using functional neuroimaging. **Methods:** An ALE (activation likelihood estimation) meta-analysis of a total of 21 studies investigating different balloon distention paradigms during either PET or fMRI was performed to demonstrate the neuroanatomical correlates of visceral pain. The ALE meta-analysis was performed using the GingerAle software version 3.0.2 and was displayed with the Mango software 4.1 on an anatomical MNI template. **Results:** Summarizing studies investigating the functional neuroanatomy of visceral pain, bihemispheric activation of the insula, the thalamus, and clusters involving the right inferior parietal lobe/postcentral gyrus as well as the left postcentral gyrus/parietal inferior lobe were observed. **Conclusions:** This ALE meta-analysis substantiates the concept of two distinguishable neuroanatomical pathways of visceral pain which are related to either the sensory-discriminative or the affective-motivational dimension of pain processing.

## 1. Introduction

Visceral pain is among the most common causes of patients seeking in-hospital treatment and affects about 20% of the general population as a chronic condition causing recurrent hospital referrals and reduced quality of life [1,2]. The International Association for the Study of Pain (IASP) defines pain as “an unpleasant sensory and emotional experience associated with, or resembling that associated with, actual or potential tissue damage” [3]. Accordingly, the sensation of pain is multi-dimensional and is related to structures representing both sensory-discriminative and affective-motivational information processing [4]. Painful or non-painful visceral sensations can be distinguished from exteroceptive sensations, e.g., cutaneous stimulation, by being characterized as dull, diffuse, and poorly localized [5]. In addition, visceral pain is oftentimes described as more unpleasant and intense and is associated with an autonomic response [6]. Conditions leading to visceral pain are diverse affecting potentially all internal organs and can be either acute, as in cholecystitis, or chronic, like in inflammatory bowel disease [1]. Experimental designs investigating visceral pain most commonly include a balloon paradigm in which the intestinal mucosa is distended with an inflated balloon leading to a painful sensation from a certain threshold [7]. The neuronal processing of visceral sensations begins with peripheral (mechano-) nociceptors which encode the stimulus intensity within the noxious and non-noxious range. Nerve fibers containing visceroafferents are normally unmyelinated and enter the spinal cord via the dorsal horn where they synapse and partly converge with other non-visceral sensory afferents. After crossing contralaterally to the anterolateral quadrant, the visceroafferents synapse with thalamic nuclei and ascend to cortical areas where the conscious sensation emerges and the behavioral response is initiated [8]. The central processing of visceral pain involves phylogenetically distinguishable brain areas with homeostatic and non-homeostatic functions. These cortical areas include the anterior cingulate cortex (ACC), the insula, the inferior parietal lobe, and the somatosensory cortex. In addition to the conscious perception of visceral pain, projections to the brainstem, the midbrain, and the hypothalamus provide a neural network of autonomic regulation [9]. There is increasing evidence that higher order cortical areas not only give rise to the conscious perception of pain but make predictions about an anticipated homeostatic state [10,11,12]. These assumptions are summarized within the concept of interoceptive inference which states that predictions about automatic homeostasis are constantly compared with the actual sensory feedback [13]. A mismatch between the prediction and the sensory feedback would lead to the emergence of a conscious sensation (predictive coding) which would induce a behavioral response (active inference) [14]. The functional neuroanatomy of brain areas involved in processing visceral pain are commonly investigated using functional magnetic resonance imaging (fMRI) or H_2_^15^O positron emission tomography (PET). There is a considerable number of studies using balloon paradigms to investigate the perception of visceral pain. Some of these studies compare visceral pain to painful somatosensory stimuli or investigate the differences between patients with chronic visceral pain, e.g., in irritable bowel disease, and healthy controls. To summarize studies on the central representation of visceral pain using either PET or fMRI, an activation likelihood estimation meta-analysis with the software GingerAle version 3.0.2 was conducted [15,16,17].

## 2. Methods

**Figure 1:**
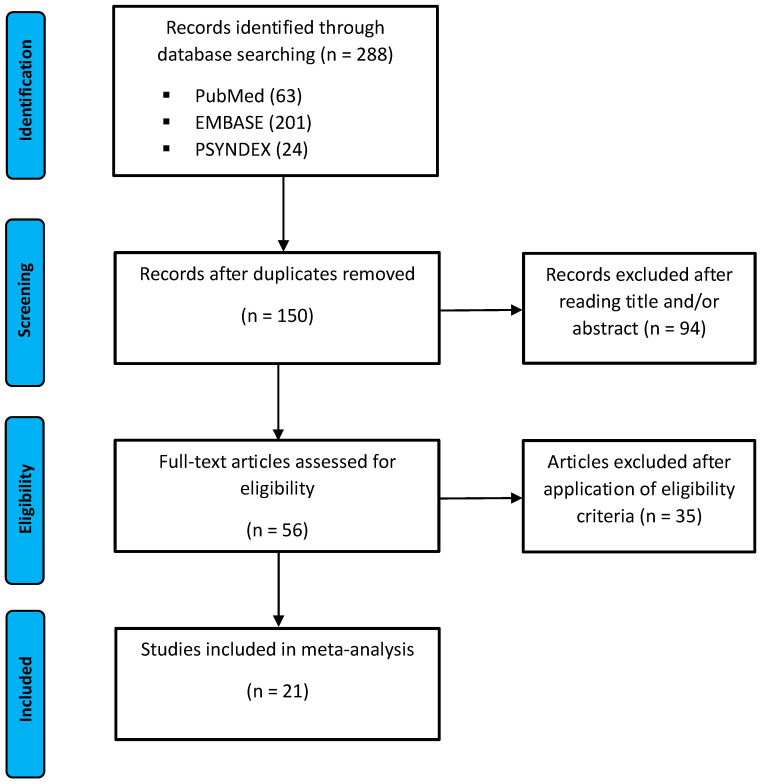
Flow chart of the study selection according to the Preferred Reporting Items for Systematic Reviews and Meta-Analyses (PRISMA).

The literature search was conducted and reported according to the Preferred Reporting Items for Systematic Reviews and Meta-Analyses (PRISMA 2020) guideline as illustrated in Figure 1 [18].

### 2.1. Data Sources and Search Strategy

The literature search was conducted across the databases PubMed, EMBASE, and PsycNet until 20 December 2024. The search strategy included the combination of each term by Boolean operator and search by proximity. The keywords and Medical Subject Heading (MeSH) terms “visceral pain” AND “fMRI” or “visceral pain” AND “PET” in title and/or abstract were used. No restrictions considering language, publication date or article type were applied. References were exported with the Citavi^©^ software version 7.0 (Lumivero, Denver, CO, USA) and duplicates were removed.

### 2.2. Eligibility Criteria

The final meta-analysis entirely consisted of prospective experimental/interventional studies investigating the neuroanatomical correlates of visceral pain induced by balloon distension of the lower esophagus, the gastric fundus or the rectum to a pre-defined or individualized threshold. Studies were considered if they used fMRI or PET as imaging modalities, and data were only extracted for healthy participants. Articles were excluded if they were reviews, letters or comments, editorials, case reports or case series and lacked information about the above-mentioned eligibility criteria or did not report foci coordinates.

### 2.3. Study Selection and Quality Assessment

The identified records were screened for eligibility by reading titles and abstracts of each article. The study selection process is illustrated in the flow chart in Figure 1. There was a total count of 288 records identified after the initial search, including 63 from PubMed, 201 from EMBASE, and 24 from PsycNet. After removal of all duplicates, 150 records were assessed for suitable reading title and/or abstract. The remaining 56 articles were then screened for eligibility. If the eligibility criteria were met, the study coordinates were extracted and included in the meta-analysis. Quality assessment was performed with the Revised Cochrane risk of bias tool for randomized trials (RoB 2.0) [19], indicating no significant bias among the included studies.

### 2.4. Data Extraction and Statistical Analysis

The x, y, z coordinates of all foci were extracted manually and written into a text file, including the reference space, study name, condition, and sample size. Conversion to the MNI (Montreal Neurological Imaging) coordinate space was performed for all foci before they were entered into the GingerAle software. The transformation into MNI space coordinates was conducted with the BioImageSuite online application (https://bioimagesuiteweb.github.io/webapp/mni2tal.html, URL accessed on 30 December 2024). The statistical analysis included studies investigating the effect of different visceral balloon distension paradigms on brain activity using fMRI or PET scans. The statistical software GingerALE version 3.0.2 (http://brainmap.org/ale/index.html, URL accessed on 20 November 2024) was used to conduct the ALE meta-analysis in an automated four step procedure. (1) Initially the data file is entered into the GingerALE software which calculates the ALE values of each voxel in a 2 × 2 × 2 mm^3^ matrix. The ALE technique estimates the uncertainty of activity in a three-dimensional space as a Gaussian normal distribution with a peak at the reported coordinate. Applying a random-effects model, a full-width half maximum (FWHM) depending on the sample size is applied to estimate the average smoothness of the entered coordinates. Thereby, a statistical map, in which each voxel corresponds to the probability that significant activation occurs, is created. (2) In a second step, a permutation test is conducted to determine the statistical significance of each ALE value. Using a Monte Carlo simulation, a random data set representing the null distribution at each voxel is calculated. The permutation test was run with 1000 permutations and an ALE map with a *p*-value for each voxel was determined. (3) Based on cluster-level inference, data were tested for significant clusters of activity above a pre-defined threshold. For the ALE map, a threshold with a *p* < 0.05 was determined. (4) The cluster-forming threshold was *p* < 0.001 for a minimum cluster size of 100 mm^3^. The ALE maps were plotted on an anatomical MNI template using the Mango software version 4.1.

## 3. Results

### 3.1. Study Selection

Finally, 21 studies were summarized in the meta-analysis. An overview on the characteristics of all included studies is provided in Table 1, Table 2 and Table 3. The included studies were published between February 2003 until March 2021 and had a prospective experimental/interventional design with 19 studies using fMRI [20,21,22,23,24,25,26,27,28,29,30,31,32,33,34,35,36,37,38] and 2 referring to PET [39,40] as imaging modality. The balloon distension paradigm was applied in the distal esophagus [20,27,30] and in the stomach [22,24,39] in 3 experiments and was conducted in the rectum for the majority of 15 studies [21,23,25,26,28,29,31,32,33,34,35,36,37,38,40]. While the majority of 18 experiments used a subjective pain threshold, a pre-determined threshold was set in 3 studies [21,26,40]. Additional investigations included a cutaneous heat stimulation [20,21], a food cold pressure experiment [25], induction of negative emotions [27], stomach filling via nasogastric tube [39], and placebo/nocebo conditions [33]. Psychological tests were conducted in several studies, including [21,22,28,29,31,32,33,35,36,38,40]. In six studies, healthy individuals were compared to patients with irritable bowel syndrome [21,23,25,26,28,37]. Summarizing the sample characteristics of all included studies, a total count of 369 individuals with a mean age of 25.8 ± 0.72 years, of which 201 (54.5%) were female, was included. Extracting the activity coordinates of healthy participants undergoing a balloon paradigm to induce visceral pain, a total number of 368 foci were considered for the ALE meta-analysis. All included studies showed significant activation in brain regions of either the somatosensory-discriminative or the affective-motivational pathway. On a qualitative level, visceral pain was perceived as more unpleasant than cutaneous noxious sensations [20] and was associated with a stronger emotional response [21]. The neuronal activity in areas of pain processing correlated with the level of anxiety [21,23,28,37] and was enhanced by the anticipation of pain [33,34] and the infusion of stress hormones [40]. In addition, one study could demonstrate that pain intensity correlates with activation in the insula and anterior cingulate cortex [31]. Although not included in the presented ALE meta-analysis, patients with irritable bowel syndrome had a similar activity pattern of both the sensory-discriminative and the affective-motivational pathway but exhibited an overall stronger neuronal activation in these areas [21,25,26,28].

### 3.2. ALE Cluster Analysis

**Figure 2:**
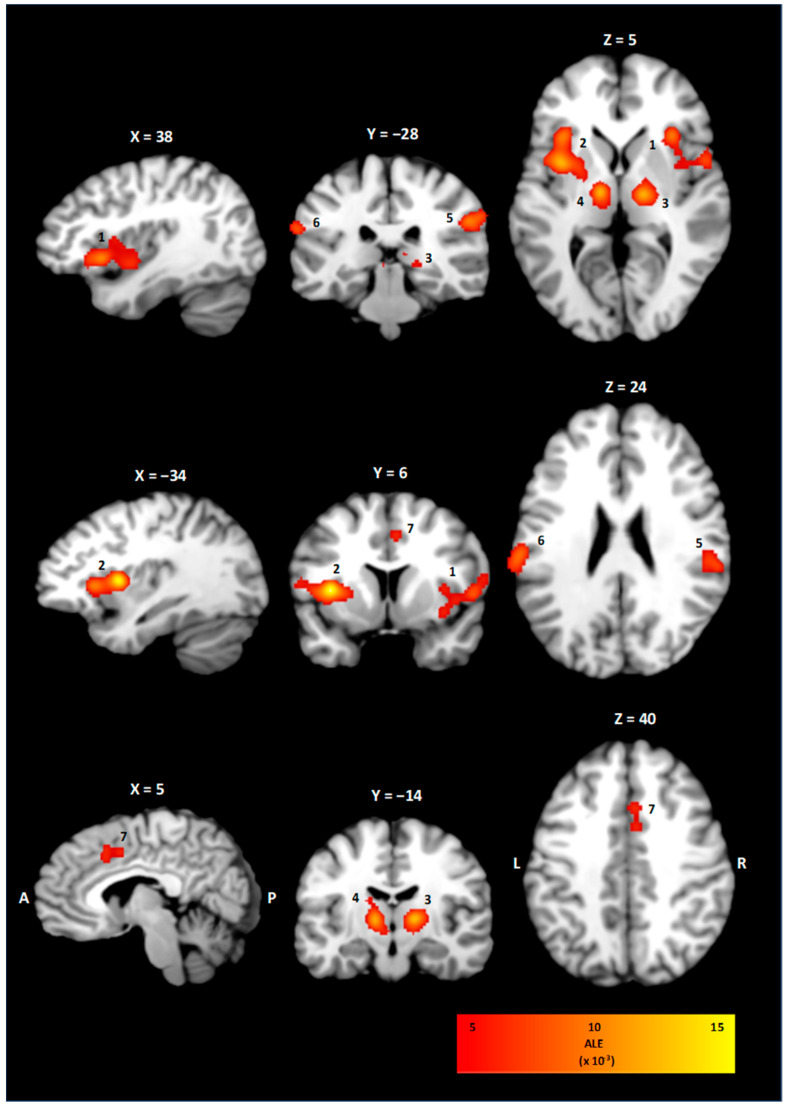
Clusters of the ALE meta-analysis in sagittal, coronal and transversal view. Statistically significant activation was found in both the right (1) and left (2) insula, the right (3) and left (4) thalamus, the right inferior parietal lobe (5), the left postcentral gyrus (6), and the right cingulate cortex (7). The ALE value of each voxel is color-coded according to the bar on the right.

The ALE meta-analysis of all included studies investigating the neuroanatomical correlates of visceral pain resulted in four statistically significant clusters of activity, as shown in Table 4 and Figure 2. On the right hemisphere, a cluster of 7024 mm^3^ with coordinates for the weighted center in (41, 11, 3) involving the insula in Brodmann’s area (BA) 13 was observed. Correspondingly, the left insula showed a statistically significant activation with coordinates for the weighted center in (−36, 6, 8). For the thalamus, clusters on the right (3592 mm^3^, 16, −14, 6) and left side (2944 mm^3^, −11, −14, 6) involving both the ventrolateral and mediodorsal thalamic nuclei were observed. Statistically significant activations in the right (2648 mm^3^, 57, −28, 29) and left inferior parietal lobe/postcentral gyrus (1592 mm^3^, −62, −24, 23) were found. The right cingulate cortex showed a statistically significant activation with a cluster size of 1344 mm^3^ and weighted center in (4, 14, 41).

**Table 1:** Studies included in the meta-analysis.

Scheme			Population		Intervention	
Author (Year)	Study Design	Imaging Modality	Sample Size (% Female)	Mean Age ± SD (Years)	Conditions	Foci
Strigo et al. (2003) [20]	Prospective experimental study	1.5 T fMRI	7 (43%)	25.8 range 19–34	Esophageal balloon distension with the balloon catheter positioned 5 cm above the lower esophageal sphincter; cutaneous heat stimulation onto the upper midline chest; two stimulus intensities low/high; stimuli were presented three times in a counter-balanced and quasi-randomized order.	24
Verne et al. (2003) [21]	Prospective experimental study	1.5 T fMRI	9 (67%)	29 ± 9	Phasic rectal balloon distension to pressures of 35 mmHg or 55 mmHg for 20 s with 20 s interstimulus control period for cycles; cutaneous pain tested with a heated water bath at three temperatures (35, 45, 47 °C); pain rating on a VAS; psychological tests with BDI, Somatic Focus (PILL), STAI, State-Trait Anger Expression Inventory, Coping Strategies Questionnaire, and the NEO-FFI.	5
Lu et al. (2004) [22]	Prospective experimental study	3 T fMRI	10 (20%)	23.6	Gastric fundus distension with an air inflated balloon; conditions of either non-painful or painful gastric distension (60–70% on a VAS rating scale); psychological test with STAI, BDI, NEO-FFI.	48
Andresen et al. (2005) [23]	Prospective experimental study	1.5 T fMRI	8 (63%)	41.3 range 27–64	Rectal balloon distensions of subliminal, liminal, and supraliminal stimulation intensities adapted to the individual perception threshold; perception rating from 1 to 6, each distension lasted for 20 s followed by a rest of 10 s.	12
Ladabaum et al. (2006) [24]	Prospective experimental study	1.5 T fMRI	18 (78%)	32 ± 6.5	Gastric balloon distension sequences of ten 45 s isobaric inflations to gastric sensation of ≥6 and <9 on a VAS scale; sequences of 45 s deflations to minimal distending pressure.	39
Hui-Song et al. (2006) [25]	Prospective experimental study	3 T fMRI	12 (100%)	23.0	Rectal balloon distension with a pressure of 20% above the pain detection threshold; foot cold pressor test with an ice water bath at 4 °C, rating on a 5-points scale.	15
Berman et al. (2008) [26]	Prospective experimental study	1.5 T fMRI	15 (100%)	36.3 ± 7.3	Rectal balloon distension, four to six stimulus sets containing 16 trials with pressures of 5, 25 or 45 mmHg.	22

Abbreviations. BDI; Beck’s Depression Inventory; fMRI, functional Magnetic Resonance Imaging; NEO-FFI, NEO-Five Factor Inventory; SD, standard deviation; STAI, State Trait Anxiety Inventory; T, Tesla; VAS, Visual Analogue Scale.

**Table 2:** Studies included in the meta-analysis.

Study ID			Population		Intervention	
Author (Year)	Study Design	Imaging Modality	Sample Size (% Female)	Mean Age ± SD (Years)	Conditions	Foci
Coen et al. (2009) [27]	Prospective experimental study	1.5 T fMRI	12 (0%)	26.0 21–31	Esophageal balloon catheter inflated to produce 40 trials of either painful or non-painful sensations; stimulation was performed under neutral or negative emotions.	11
Elsenbruch et al. (2009) [28]	Prospective experimental study	1.5 T fMRI	12 (100%)	31.4 ± 2.3	Rectal balloon distension, staircase increments of 2–10 mmHg until a subjective threshold of 5 on a 6-point scale was reached; ratings on HADS, STAI-S, and SCL-90-R.	6
Moisset et al. (2010) [29]	Prospective experimental study	1.5 T fMRI	11 (100%)	38.4 ± 3.1	Rectal balloon distension until a subjective threshold of either 2 (non-painful stimulus) or 5 (painful stimulus) on a 6-point scale; psychological test with PAS and short-form McGill questionnaire.	21
Lu et al. (2010) [30]	Prospective experimental study	3 T fMRI	14 (64%)	23.9 ± 3.9	Esophageal balloon distension until 60–70% of subjective pain intensity, administration of normal saline during control condition; during placebo conditions, participants were told to receive an opioid intravenously; ratings on PCS, VAS, and McGill pain questionnaire.	27
Benson et al. (2011) [31]	Prospective experimental study	1.5 T fMRI	30 (50%)	25.75 ± 6.1	Rectal balloon distensions until an individual pain threshold of 5 on a 6 points scale; eight phases of distension alternated with eight phases without distension and a duration of 31 s for each condition; auditory cue before distension to assess anticipation; rating with HADS and VAS.	17
Geeraerts et al. (2011) [39]	Prospective experimental study	PET	14 (29%)	26.3 ± 1.8	Gastric fundus balloon distension and continuous and stepwise infusion until individualized abdominal discomfort threshold; comparison between intragastric volumes of balloon distension and continuous or stepwise meal infusion; rating on satiation or upper abdominal sensation scale.	6
Smith et al. (2011) [32]	Prospective experimental study	3 T fMRI	14 (100%)	n.a.	Rectal balloon distension; five repetitions of four conditions consisting of no stimulus, subliminal stimulus, liminal stimulus and painful stimulus for 40 s each; rating with HAD, PHQ-15, and VAS.	34

Abbreviations. HADS, Hospital Anxiety and Depressions Scale; n.a., not available; PCS, Pain Catastrophizing Scale; PHQ-15, Patient Health Questionnaire; SCL-90-R, Symptom Checklist revised; SD, standard deviation; STAI, State Trait Anxiety Inventory; T, Tesla.

**Table 3:** Studies included in the meta-analysis.

Study ID			Population		Intervention	
Author (Year)	Study Design	Imaging Modality	Sample Size (% Female)	Mean Age ± SD (Years)	Conditions	Foci
Schmid et al. (2013) [33]	Prospective experimental study	3 T fMRI	36 (50%)	29.7 ± 1.8	Rectal balloon distention until a subjective pain threshold between 5 and 6 on 6-point scale; two groups of intravenous infusion of saline/spasmolytic drug (placebo) or saline/opioid antagonist (nocebo); investigation of anticipated effect; rating on HADS, BDI and baseline cortisol levels.	15
Theysohn et al. (2014) [34]	Prospective experimental study	3 T fMRI	30 (50%)	34.7 ± 3.2	Rectal balloon distension until an individualized pain threshold following a visual stimulus, counterbalanced by visually cued resting periods; participants were told that they would receive an analgesic drug or an inert substance; rating on a VAS scale.	10
Gramsch et al. (2014) [35]	Prospective experimental study	3 T fMRI	24 (54%)	28.8 ± 8.5	Rectal balloon distension, conditioning paradigm with visual cues to measure distension and anticipation-related neural activation; pain threshold set at subjective pain rating between 5 and 6 on a 6-point scale; ratings on VAS and HADS.	11
Icenhour et al. (2016) [36]	Prospective experimental study	3 T fMRI	40 (53%)	26.00 ± 3.27 range 20–32	Rectal balloon distensions of high or low intensity paired with visual cues to assess pain and anticipation-related neural activation; pain threshold defined as low intensity for a rating of 4 and high intensity between 5 and 6 on a 6-point scale; rating on VAS, HADS, and STAI.	6
Tanaka et al. (2016) [40]	Prospective experimental study	PET	16 (0%)	22.8 ± 2.5	Rectal balloon distensions at mild (20 mmHg), intense (40 mmHg) compared to baseline and no distension (0 mmHg); effect of CRH or saline intravenously on brain activation was assessed; plasma ACTH, serum cortisol and plasma noradrenaline levels at each stimulation.	12
Guleria et al. (2017) [37]	Prospective experimental study	3 T fMRI	10 (0%)	28.5 range 26.5–31.5	Rectal balloon distensions to an individualized pain threshold; comparison between healthy controls and patients with IBS.	5
Icenhour et al. (2021) [38]	Prospective experimental study	3 T fMRI	27 (44%)	25.7 ± 1.0	Rectal balloon distensions in different visual contexts; interoceptive cues were followed by visceral pain as conditioned stimulus; ratings on VAS, HADS, TICS, and STAI.	22

Abbreviations. BDI, Beck’s Depression Index; CRH, Corticotropin-Releasing Hormone; HADS, Hamilton Anxiety and Depression Scale; SD, standard deviation; STAI, State-Trait Anxiety Inventory; T, Tesla; TICS, Trier Inventory for Chronic Stress.

**Table 4:** Location of clusters with statistically significant brain activation and coordinates for the weighted center for all included studies.

Cluster	Brain Region	BA	x	y	z	Volume (mm^3^)	ALE (×10^−3^)
1	Right Insula	13	41	11	3	7024	27.0
2	Left Insula	13	−36	9	7	6080	37.5
3	Right Thalamus	-	16	−16	6	3592	30.1
4	Left Thalamus	-	−11	−14	6	2944	30.1
5	Right Inferior Parietal Lobe	40	57	−28	29	2648	26.2
6	Left Postcentral Gyrus	40	−62	−24	23	1592	23.1
7	Right Cingulate Cortex	32	4	14	41	1344	16.4

Abbreviations. ALE, activation likelihood estimation; BA, Brodmann area.

## 4. Discussion

This ALE meta-analysis demonstrates the functional neuroanatomy of visceral pain by synthesizing studies using either PET or fMRI during balloon paradigms in healthy individuals. The cluster analysis of all included studies showed statistically significant activation for structures related to the sensory-discriminative pathway of pain processing, like the somatosensory cortex, the inferior parietal lobe, and the ventrolateral thalamic nuclei. In addition, statistically significant activation in areas engaged in emotional and autonomous processing involving the insula and cingulate cortex were observed.

The activation of neural structures representing central correlates of visceral pain is related to distinct pathways conveying sensory information of inner organs. The cell bodies of the first order neurons of these pathways are located in remote ganglia distinguishing them from the enteric or intrinsic nervous system with its cell bodies within the gut wall. Their sensory information is encoded by either low or high threshold mechanoreceptors and is transmitted via myelinated Aδ or unmyelinated C fibers [41].

Vagal sensory neurons, which receive their input from low threshold, slowly adapting receptors, are unmyelinated and carry information from thermal, mechanical, and chemical stimuli. Vagal mechanoreceptors respond to intraluminal pressure or muscle contraction and cover the esophagus, stomach, and the proximal colon [42]. Although there is convergence with other pain pathways on the level of the spine and brainstem, the primary goal of these vagal afferents which ultimately project to the solitary tract nucleus appears to be visceral homeostasis and not pain perception [43].

Spinal sensory neurons receive input from both high and low threshold receptors and convey mainly via more rapidly conducting Aδ fibers. These spinal neurons can be divided into thoracolumbar (splanchnic) afferents innervating the entire gastrointestinal tract and sacral (pelvic) afferents receiving input from the rectum and surrounding structures of the pelvis. While pelvic visceroafferents can be regarded as the lumbosacral equivalent of vagal sensory innervation and mainly contribute to autonomous homeostasis, the splanchnic nerves are related to visceral pain sensation [44].

**Figure 3:**
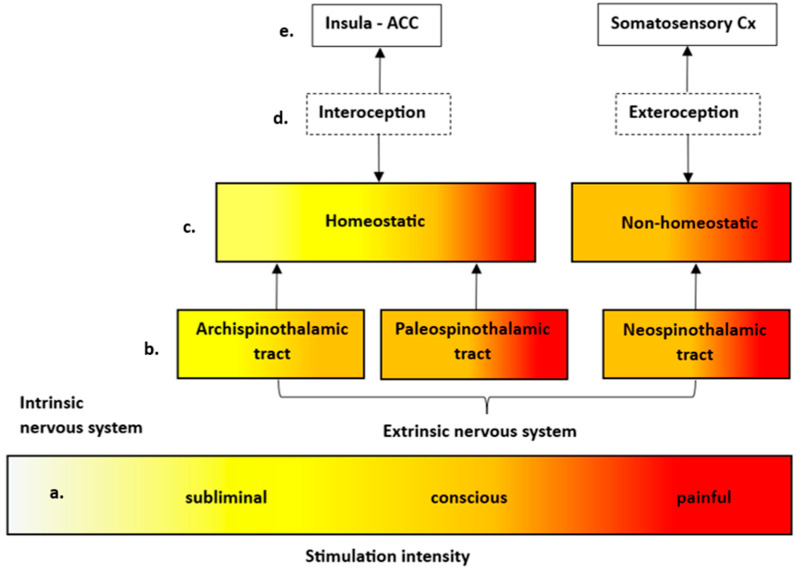
Simplified illustration of the **a.** stimulation intensity with its representation in **b.** phylogenetically distinguishable spinothalamic tracts and its relation to **c.** either homeostatic or non-homeostatic functions, **d.** interoception vs. exteroception with its associated **e.** key neuroanatomical structures.

Phylogenetically, a distinction between the neospinothalamic, the paleospinothalamic, and the archispinothalamic tract can be made (Figure 3) [10,45]. The neospinothalamic tract, as the phylogenetically youngest pathway, consists of the lateral spinothalamic tract whose first order neurons synapse in Rexed layer I of the dorsal horn. After decussating contralaterally, they ascend within the anterolateral quadrant to the ventroposterolateral and ventroposteroinferior thalamic nuclei from where they eventually project to the primary somatosensory cortex [46]. The lateral spinothalamic tract receives input mainly from A-fibers and gives rise to a rather sharp, more easily localized pain [45]. Regarding the results of the presented ALE meta-analysis, the observed activations of the somatosensory cortex, the inferior parietal lobe, and the ventrolateral thalamic nuclei can be interpreted as the central correlates of sensory-discriminative pain perception [47]. On a qualitative level, the sensation transmitted through the lateral spinothalamic tract differs from the characteristic perception of visceral pain which is generally described as being more vague, diffuse, and difficult to localize. This may be explained by the fact that all included studies involved a distension paradigm with a subjective pain or pre-defined pressure threshold at which the luminal wall was distended. Thereby, it is likely that the distension did not only lead to visceroafferent stimulation but may also irritate the visceral peritoneum causing a somatic rather than visceral pain sensation [48]. Activation in the inferior parietal lobe is related to sensorimotor integration of different sensory input thereby linking perception and action by sending motor commands to posterior parts of the frontal lobe [49].

The paleospinothalamic tract is mainly related to the anterior spinothalamic pathway which receives information from Aδ and C fibers with cell bodies in the dorsal root ganglia. These neurons in Rexed layer II project to nerve cells in layers IV to VIII which then decussate to the anterolateral quadrant. From there, projections are sent to the reticular formation and the periaqueductal grey (spinoreticular tract), the tectum (spinotectal tract), and to the mesencephalon (spinomesencephalic tract). By projecting to the limbic system and the hypothalamus, these fiber tracts provide the sensory input for the emotional processing of visceral pain and the adaptive autonomous response [50].

As the phylogenetically oldest pathway, the archispinothalamic tract mediates visceral, emotional, and autonomic reactions to painful stimuli. Its first order neurons with cell bodies in the dorsal root ganglia project to Rexed layers IV and VII in the spinal cord which send afferents to the midbrain reticular formation and the periaqueductal grey [46]. From there, collaterals are sent to the limbic system and the hypothalamus enabling autonomous and emotional processing of subliminal or consciously perceived visceral stimulation. In essence, visceral pain being described as more diffuse, vague, and difficult to localize may be predominantly represented by the phylogenetically older paleospinothalamic and archispinothalamic tracts, while a sharp and well-localized pain sensation involving the peritoneum is transmitted via the neospinothalamic tract [9,45]. Its central projections diverge at the thalamic level with the lateral nuclei projecting to the primary and secondary somatosensory cortex (lateral system) and the medial thalamic nuclei sending afferents to the limbic system and areas of autonomous control (medial system). Therefore, based on its relation to different thalamic nuclei, the neospinothalamic tract is equivalent to the medial system, while the paleo- and archispinothalamic tract are associated with the lateral system [8].

From a neurophysiological perspective, a distinction between the observed neuroanatomical activations can be made depending on whether their goal is related to either homeostatic or non-homeostatic functions. Visceral stimuli associated with a homeostatic response are processed within the archispinothalamic and paleospinothalamic pathway which induce an unconscious or conscious response of the effector system. The awareness of usually unconscious visceral stimuli is referred to as interoception and is anatomically organized via visceroafferents projecting to the insula and anterior cingulate cortex (ACC) [11]. It is hypothesized that the insula provides homeostatic set-points against which the ascending sensory input is compared. Its activation would then result from a divergence or mismatch from these homeostatic states, which would lead to afferent signals being sent to the ACC. While the insula gives rise to the feeling of a disbalance of physiological states, the ACC attributes a motivational value, thereby creating basic emotions which drive future behavior towards an anticipated goal [51]. This is conceptualized within the somatic marker theory of consciousness, which states that emotions require brain regions engaged in the regulation of internal states so that feelings can help in guiding future behavior [52].

Anatomically, the insula is divided by the central sulcus into an anterior and posterior part. Stimulation of the anterior insula has been shown to evoke viscerosensory perception, whereas induced activation of the posterior insula is associated with somatosensory symptoms [53]. Accordingly, the anterior agranular cortex of the insula is connected to the limbic, the orbitofrontal, and the cingulate cortex, while the posterior part projects to the amygdala and the dorsal thalamus.

Functionally, the insula can be divided into four regions which integrate incoming information from posterior to mid-anterior [54]. Anterior-ventral areas of the insula show connections with the ACC and provide a socio-emotional valance to visceral perceptions.

While central parts of the insular cortex are related to olfactogustatory perceptions, the mid-posterior insula integrates incoming visual and auditory information, which may induce an adaptive motor response.

The cingulate cortex can be functionally divided into four posterior to mid-anterior areas. The anterior part, as part of the ventral or “what” stream, is involved in emotional salience and integration of incoming visceral sensations [55]. It encodes autonomic states which may help guiding actions towards or away from an anticipated goal [56,57]. The medial cingulate cortex, which is connected to the posterior cingulate cortex (PCC), is engaged in behavioral decisions and is related to visuospatial orientation. The retrospenial cortex contributes to the consolidation of memories by sending input to hippocampal areas [58].

As previously mentioned, the awareness of usually unconscious states is referred to as interoception and can be embedded within the concepts of predictive coding and active inference [43,51]. Accordingly, visceromotor areas (VMA), like the ACC, generate predictions about future sensory events which are compared with the actual sensory feedback. A mismatch between these predictions and the feedback from visceral afferents causes a prediction error which results in the actually perceived sensation. This is structurally organized within visceromotor efferents not only sending motor commands to the intestinal wall but providing an efference copy for viscerosensory cortical areas [59]. In case of a mismatch, predictions generated by VMA are updated to match the actual sensory feedback. This process of neuronal adaptation referred to as predictive coding could be related to the activation of both the insula and cingulate cortex, which provide homeostatic set-points against which the feedback of visceral afferents is compared [13]. In addition to the emergence of a painful sensation, the prediction error induces a response of the effector system to correct for the detected disbalance. This control and regulation system includes an autonomic reflex circuit for the autonomic pathway and projections to higher motor areas for the somatosensory system. Theoretically, the induction of a behavioral response is related to the framework of active inference, which states that prediction errors cause the effector system to seek for a resolution of the occurred mismatch. This is implemented with the ACC being involved in response selection by sending afferents to higher-order motor areas [60].

Therefore, interoception as the perception of usually unconscious inner states can be viewed as a basic consciousness which guides the individual’s behavior towards regaining autonomic homeostasis. Besides its immediate behavioral consequences, the affective-motivational pathway may lead to long-term alterations in the processing of sensory information via projections to the perirhinal/entorhinal cortex [58].

This may explain the interindividual differences in visceral pain perception, which is cognitively and emotionally modulated based on previous experiences. In addition, the concept of gain control in interoceptive inference relates to the finding that the emergence of these sensations is influenced by the current emotional and attentional state which may or may not contribute to the awareness of a mismatch between predictions and sensory feedback [61]. However, this subjectiveness in the experience of pain may be associated with significant variability of the neuroanatomical correlates activated during each study. Most studies included young healthy individuals whose peripheral and central neuronal processing may differ significantly from patients with psychiatric comorbidities and functional disorders, e.g., irritable bowel syndrome [21,25,26,37]. Another source of heterogeneity may be an unbalanced male to female ratio among studies leading to gender-specific variability. Methodologically, the balloon paradigms differed with regards to the stimulated organ and the intensity threshold, which was either pre-determined or set to a subjective pain threshold. Although data was only extracted for healthy individuals undergoing an intestinal balloon distension, some studies included trials of anticipated pain or placebo effects which may have influenced the condition of interest. The modulating effect of the current emotional state was investigated by some studies indicating a significant influence on the neural activation during visceral pain [27,28,31,32,33,35,36,38]. Given the presumed influence of the affective state on pain perception, it would be interesting to know whether pharmacological treatment or psychotherapy may lead to measurable neuronal changes during pain processing. Future research may also focus on the specific neuroanatomy of patients with functional disorders to provide a better understanding of the complex interplay between psychological variables and pain perception.

## 5. Conclusions

This ALE meta-analysis demonstrates that the sensation of visceral pain can be described on a sensory-discriminative and affective-motivational level. These perceptual dimensions are related to neuroanatomical substrates that allow for the interoception of usually unconscious inner states and may thereby enable the individual to adapt its behavior towards regaining autonomic homeostasis. Despite some heterogeneity among the included studies considering biometric characteristics, the applied balloon paradigm, and the imaging modality, the present work points out two distinguishable pathways of pain processing. The activation of the lateral thalamic nuclei, the postcentral gyrus, and the inferior parietal lobe is related to the sensory-discriminative network of pain processing, which gives rise to a rather sharp more easily localized sensation. Activity in the medial thalamic nuclei, the insular, and the cingulate cortex is associated with a more diffuse, difficult-to-localize pain perception and may induce autonomic homeostatic responses even if the stimulus is not perceived consciously. Both pathways can be distinguished regarding sensory qualities, functional neuroanatomy, physiological functions, and phylogenetical origin.

Theoretically, the perception of visceral sensations and the behavioral response can be explained within the framework of predictive coding and active inference. The observed neuroanatomical correlates of pain perception involving both the limbic and the somatosensory system substantiate the clinically observed individual differences in pain perception, which can be modulated by previous experiences and the current emotional and attentional state. Further research may focus on the differences in pain processing in patients with functional disorders and chronic visceral pain to gain a better understanding of its specific functional neuroanatomy.

## Data Availability

No new data were created or analyzed in this study.

## Abbreviations

ACC	Anterior cingulate cortex
ALE	Activation likelihood estimation
BA	Brodmann area
fMRI	Functional magnetic resonance imaging
FWHM	Full width half maximum
IBS	Irritable bowel syndrome
MNI	Montreal Neurological Imaging
PCC	Posterior cingulate cortex
PRISMA	Preferred Reporting Items for Systematic Reviews and Meta-Analyses
VMA	Visceromotor areas
